# Prevalence and intensity of catastrophic health care expenditures in Iran from 2008 to 2015: a study on Iranian household income and expenditure survey

**DOI:** 10.1186/s12939-018-0743-y

**Published:** 2018-04-13

**Authors:** Vahid Yazdi-Feyzabadi, Mina Bahrampour, Arash Rashidian, Ali-Akbar Haghdoost, Mohammadreza Akbari Javar, Mohammad Hossein Mehrolhassani

**Affiliations:** 1 Social Determinants of Health Research Center, Institute for Futures Studies in Health, Kerman University of Medical Sciences, Kerman, Iran; 2 Health Care Services Management Department, Health Services Management Research Center, Kerman University of Medical Sciences, Kerman, Iran; 3 Department of Health Management and Economics, School of Public Health, Tehran University of Medical Sciences, Tehran, Iran; 4 Regional Knowledge Hub, and WHO Collaborating Centre for HIV Surveillance, Institute for Futures Studies in Health, Kerman University of Medical Sciences, Kerman, Iran; 5 Modeling in Health Research Center, Institute for Futures Studies in Health, Kerman University of Medical Sciences, Kerman, Iran; 6 Medical Informatics Research Center, Institute for Futures Studies in Health, Kerman University of Medical Sciences, Kerman, Iran

**Keywords:** Health expenditure, Financial equity, Health policy, Iran

## Abstract

**Background:**

Households exposure to catastrophic health expenditure is a valuable measure to monitor financial protection in health sector payments. The present study had two aims: first, to estimate the prevalence and intensity of catastrophic health expenditures (CHE) in Iran. Second, to investigate main factors that influence the probability of CHE.

**Methods:**

CHE is defined as an occasion in which a household’s out-of-pocket (OOP) spending exceeds 40% of the total income that remains after subtraction of living expenses. This study used the data from eight national repeated cross-sectional surveys on households’ income and expenditure. The proportion of households facing CHE, as a prevalence measure, was estimated for rural and urban areas. The intensity of CHE was also calculated using overshoot and mean positive overshoot (MPO) measures. The factors affecting the CHE were also analyzed using logistic random effects regression model. We also used ArcMap 10.1 to display visually disparities across the country.

**Results:**

An increasing number of Iranians has been subject to catastrophic health care costs over the study period in both rural and urban areas (CHE = 2.57% in 2008 and 3.25% in 2015). In the same period, the overshoot of CHE and the mean positive overshoot ranged from 0.26% to 0.65% and from 12.26% to 20.86%, respectively. The average absolute monetary value of OOP spending per month has been low in rural areas over the years, but the prevalence of CHE has been higher than urban areas. Generally put, rural settlement, higher income, receiving inpatient and outpatient services, and existence of elderly people in the household led to increase in CHE prevalence (*p* < 0.05). Interestingly, provinces with more limited geographical and cultural accessibility had the lowest CHE.

**Conclusions:**

According to the findings, Iran’s healthcare system has failed to realize the aim of five-year national development plan regarding CHE prevalence (1% CHE prevalence according to the plan). Therefore, revision of financial health care protection policies focusing on pre-payments seems mandatory. For instance, these policies should extend the interventions that target low-income populations particularly in rural areas, provide more coverage for catastrophic medical services in basic benefit packages, and develop supplementary health insurance.

## Background

Healthcare financial protection, as one of the building blocks of universal health coverage (UHC), has been under increasing attention over the recent years [1, 2]. To ensure such a protection, health policy makers resort to some measures like risk spreading, risk pooling, equitable provision of resources, and application of adequate and sustainable resources in financing of health systems [3]. Beside other financial indices, catastrophic health expenditures (CHE) are used to evaluate and monitor financial protection in health systems across the world. There are various methods to estimate the proportion of households facing CHE [4]. One of the most common methods is the definition of World Health Organization (WHO) of CHE. According to this definition, household’s health expenditures are catastrophic when they are equal to or more than 40% of household expenditure after subtracting the subsistence costs [5]. Overall, this index represents the out-of-pocket (OOP) spending (the amount paid at the point of service delivery) that exceeds a certain amount of households’ budget [6].

Increased OOP can cause economic hardship, particularly in developing countries [6, 7]. As OOP increases for healthcare services, risk spreading and pooling fades away and resource allocation becomes unfair. As a result, vulnerable individuals experience financial hardship and households face CHE [8]. The OOP can also be a reflection of social inequalities and lack of suitable social protection, especially efficient insurance mechanisms [9]. The OOP, as the most inefficient mechanism for health financing, has the greatest burden on the poor and is related to higher risk of household’s impoverishment due to CHE [10]. It also can lead to impoverishment and loss of income because of selling household assets to finance health service needs [11]. It may also result in increased pressure on households’ budget, delayed treatment due to payment inability, distrust of health insurance systems, and rapid poverty growth in a society. Although decreased OOP spending can reduce CHE and impoverishment, this effect depends on the type and practicality of purposeful interventions to reduce direct payments.

Previous studies have shown that the rate of CHE ranges from 0.8% to 6.3% in developing countries such as Argentina, Colombia, Mexico, and Thailand [5]. A study in Iran [12] showed that the CHE rate ranged from 2.2% to 2.5% between 2003 and 2007. Over the past decades, Iran’s health system has taken-up some major policies to improve fair financing and reduce OOP payments. Bed insurance, free treatment of vehicle accident victims, establishment of family physician and rural insurance programs, establishment of board of trustees in educational hospitals, full-time geographic programs for physicians, and recent Iran’s health transformation program (IHTP) conducted in early 2014 are examples of such protecting policies. Moreover, in 4th and 5th national development plans, reduction of OOP proportion of total health expenditures (THE) from 50% to 30% and reduction of households’ exposure to CHE to 1% were set as the targets to protect the health care financially [13, 14]. These targets in 6th national development plan were justified from 58% to 25% and from 6% to 1% for OOP proportion of THE and households’ exposure to CHE, respectively [15].

Several cross-sectional studies are conducted to measure CHE among different groups [16] and in some local areas [17–19] which show different rates. The majority of these studies are conducted in a small area or a short period of time that made it difficult to monitor the index for whole the country. While, only a few studies have been carried out on national data [12, 20]. Hence, there is no evidence of CHE trend over a relatively long period of time in Iran, particularly after implementation of IHTP which aimed to improve financial protection of Iran’ health system through reduction in OOP spending [21]. Moreover, there is a lack of research on rural vs. urban areas differences and disparities across provinces in this regard. Such research on rural-urban differences and across provinces can help policy makers specify interventions and improve the CHE by focusing on at risk settings and improving equity in financial accessibility through filling gap between areas. The main aim of the present study was to estimate the prevalence and intensity of households’ exposure to CHE in rural and urban areas in Iran. We also aimed to determine household characteristics effects on CHE from 2008 to 2015 and display variation of CHE disparities across provinces in that period.

### A brief of health care financing and IHTP in Iran

Iran’ health system is divided into two wide areas of PHC network and medical services that includes public and private inpatient and outpatient services. PHC is more comprehensive in rural areas with an organized referral system supported by rural family physician program and rural health insurance scheme [22]. Health care financing in Iran is a mixed-financing system in which three major types of financing (i.e. general government budget, social health care insurance payments, and household OOP payments) play a role. There is also private health care insurance program that provides secondary coverage for those already insured by social insurance. General government budget mainly focuses on PHC network and some special diseases (e.g. hemophilia, kidney replacement therapies, renal kidney failure, and thalassemia). Governmental budget also invests on infrastructure of public hospitals and production of medicines [23, 24].

According to a national survey conducted in 2010, basic and supplementary health insurances cover 83.15% and 12.46% of the population in Iran, respectively [25]. Of course after implementing IHTP, the percentage of uninsured population in Iran was highly decreased and a remarkable number of uninsured (about 10 million people) were covered by Iran’s health insurance [21]. Social healthcare insurance has a fragmented structure in Iran, making some challenges in risk and resource pooling strategies. It mainly focuses on medical services that include ambulatory, diagnostic, and hospital services. The main public health insurance organizations with high rates of coverage in Iran are as follows: Social Security Organization (SSO), Iran’s Health Insurance Organization, Armed Forces Medical Services Insurance Organization, and Imam Khomeini Relief Foundation Health Insurance. Moreover, there are several private health insurance funds such as those of petroleum industry and banks. Public hospitals, which are managed by medical universities, provide a considerable proportion of inpatient services. Besides purchasing services from other providers, the SSO and private health care insurance funds provide direct medical services through their own hospitals and clinics as well. These can be named for the flaws of health system in the country: high OOP spending, quality of care challenges (especially in inpatient services in public sectors), fragmented health care services, and inefficient referral system (especially in urban areas). According to reports, the OOP spending before the implementation of IHTP claimed of about 50% of total health expenditures [26].

IHTP is comprised of a series of reforms that follow a step-wise process. One of the main objectives of this program is reduction of OOP and improvement of health care in order to move towards universal health coverage. The first step of this program was launched in May 2014 that focused on improvement of infrastructures and reduction of OOP spending in public hospitals. The second step of this program initiated in October 2014 that aimed to revise medical tariffs rate based on relative value units (RVUs) in order to eliminate informal payments received by providers. This matter resulted in rapid growth of tariffs among different medical specialties. Some of the main interventions of this program were as follows:

- Free access to basic health insurance for all uninsured individuals
- Reduction of OOP for inpatient services in public hospitals
- Improvement of financial protection of patients with special diseases
- Adoption of promotional policies for retention of specialist and general practitioners in deprived areas
- Adoption of interventions such as recruitment of more specialists to improve quality of services delivered in hospitals
- Improvement of quality of outpatient services in polyclinics
- Improvement of hospital amenities, lodging services, and hoteling services in hospitals
- Balancing of medical tariffs of some procedures (e.g. delivery vs. cesarean section)
- Standardization of medical tariffs so that they increase and reach to realistic rates [21] IHTP also contains some efforts to improve services in PHC networks that focus on non-communicable diseases prevention and care and their extension to marginalized areas.

## Methods

### Samples and data source

In a retrospective descriptive study that spanned from 2008 to 2015, required data were obtained from eight national repeated cross-sectional surveys on annual income and expenditure surveys. These surveys are annually run by Iran Statistics Center (ISC). These surveys include 1) social characteristics of household members; 2) housing characteristics, living facilities, and assets; 3) household food and non-food expenditures; and 4) household income. In the surveys, households were selected based on a three-stage stratified random cluster sampling method. The census areas are classified and selected at the first stage. At the second stage, the urban and rural blocks are selected and the selection of ‘households is done at the third stage. Samples were selected from both urban and rural areas [27]. Households were weighted by ISC according to differences in urban and rural population ratio (two-thirds of the population live in urban areas). In this manner, the proportion of households within each cluster, rural/urban and provinces were firstly determined; then sampling weights were calculated for each household based on the inverse of the likelihood of being sampled (i.e. $\frac{N}{n}$, where N is number populations and n is number samples). After exclusion of households whose food expenditure was not reported, final sample size ranged from 36,772 to 39,008 in the studied years. The sample size varied from 18,166 to 19,739 and from 18,502 to 19,338 for rural and urban areas, respectively (Table 1).

### Measurements

The method proposed by WHO was used to calculate the CHE annually. In this method, financial catastrophe occurs when household’s OOP spending equals or exceeds 40% of its capacity to pay (CTP) [28]. Since the recall period for each item in household expenditure questions was different (ranging from one month to one year) in surveys, month was chosen as the basis of analyses. In addition, as each survey took more than one month to complete, expenditures were modified by ISC using consumer price index.

In order to measure CHE, one should firstly calculate households’ capacity to pay, as “the effective income (total household expenditure) minus the basic living needs (subsistence spending) adjusted for household size”. Simply put, CTP equals households’ non-food expenditure [5].

Given the economy scale of household consumption, equivalence scale was used instead of actual household size in CTP calculation:

$$ {eqsize}_h={hsize}^{\upbeta} $$

Where “hsize” is actual household size and *eqsize*_*h*_ is equivalence size of the household. Based on a similar study on 59 countries, β was considered to be 0.56 [5].

One also needs to calculate poverty line (PL), which is a minimum spending to protect the basic needs (i.e. subsistence spending). The PL was calculated based on households’ food share. For this end, mean of absolute food expenditure was calculated for households whose food share of total household expenditure ranged from 45 to 55%. The PL was separately measured for rural and urban households as shown below:

$$ {eqfood}_h=\frac{food_h}{eqsize_h} $$

$$ \mathrm{pl}=\frac{\sum {w}_h\times {eqfood}_h}{\sum {w}_h}; food\ 45<{foodexp}_h< food55 $$

Where *w*_*h*_ is sampling weight of households and *pl* stands for poverty line.

Moreover, subsistence spending of household (*se*_*h*_) was calculated as:

$$ {se}_h= pl\times {eqsize}_h $$

Some households reported that their food expenditure was lower than subsistence spending (*se*_*h*_ > *food*_*h*_). This could be due to the fact that reported food expenditure in surveys did not consider food subsidies, coupons, self-production, and other non-cash means of food consumption. In situations like this, the food expenditure is lower than the estimated poverty standard for that country, so the CTP or household non-subsistence spending equals [28]:

$$ {ctp}_h={\mathit{\exp}}_h-{food}_h\kern0.75em \mathrm{if}\kern0.5em {se}_h>{food}_h $$

or

$$ {ctp}_h={\mathit{\exp}}_h-{se}_h\ \mathrm{if}\kern0.5em {se}_h\le {food}_h $$

Where *ctp*_*h*_ is CTP and *exp*_*h*_ is the total expenditure. Then the OOP (payment for health at the point of service delivery) is divided by CTP and is called as “burden of household health payments”. It is calculated as follows:

$$ { oop ctp}_h=\frac{oop_h}{ctp_h} $$

Then, if the above equation is greater than or equal to 0.4 (*ifoopctp*_*h*_ ≥ 0.4), the household experience CHE.

The percentage of households experiencing catastrophic payments, named as catastrophic head count (HC), is estimated as follows:

$$ H=\frac{1}{N}{\sum}_{i=1}^N{E}_i $$

Where N equals the sample size. Regarding E, If OOP of household *i* is equal or greater than the threshold, E = 1; otherwise, it equals zero. HC measures the proportion of households whose OOP is above the threshold, but it does not measure the degree by which payments exceed the threshold. The intensity of CHE is calculated using two measures inclding overshoot and mean positive overshoot (MPO) measures. Overshoot shows the average degree by which OOP payments exceed the threshold (Z). The threshold in this article is 0.4. Thus overshoot gives an indication of how much OOP payments exceed the threshold (i.e. 0.4). The overshoot was calculated based on the following formula [29]:

$$ {O}_i={E}_i\left(\left(\frac{oop_h}{ctp_h}\right)-Z\right) $$

Then, a household is said to have experienced catastrophic payments when$\frac{oop_h}{ctp_h}$exceedsthe threshold. The average overshoot is [29]:

$$ O=\frac{1}{N}{\sum}_{i=1}^N{O}_i $$

Another measure used to calculate i1ntensity of CHE is MPO which means the payment in excess of the threshold, averaged over all households exceeding that threshold. Thus, MPO is the overshoot divided by HC [29]:

MPO = $$ \frac{O}{H.} $$

#### Outcome and independent variables

We also examined that what factors may affect CHE. Thus, we used a binary outcome variable (with value 1 indicating a household with catastrophic expenditure, and 0 without catastrophic expenditure). Independent variables were selected on the criteria of the frequency with which they were used in past studies and availability of data. Thus, socio-demographic characteristics that were used to investigate the associated of CHE were as follows: place of residence (rural-urban dichotomy), gender of household head, inpatient healthcare during the preceding 12 months, outpatient care during the preceding month, existence of elderlies living in the household during the preceding 12 months, and income level.

### Data analysis

To convert Rial (Iran’s currency) to international dollar (PPP), American dollar (USD) exchange rate was first obtained from the Central Bank of Iran for each year [30]. Then, PPP conversion factor was obtained from the World Bank [31] and the equivalence of 1 PPP in terms of Iran’s Rial was estimated for each year.

The prevalence of CHE, its overshoot, and MPO were separately calculated for rural and urban areas. The confidence interval (CI) was also reported for CHE prevalence in study years.

To examine factors affecting the CHE, as a binary outcome variable, we should use a logistic regression model. However, when observations in cross-sectional data are selected based on clusters, a cluster effect can be anticipated within clusters. Thus, the ignoring of clustering will usually increase the resulting variance of the estimators as the independence between observations is violated due to the correlation within clusters. so that estimating standard errors is not valid and would be biased. Here, the households are clustered within provinces and therefore adjustment of the logistic model for the clustering effect in estimating the standard errors of the parameter estimates is required. Thus, we used a logistic random effects regression model for adjusting the cluster effect of province variable. In the first phase, a bivariate analysis was conducted on the data. Then, variables with a significance level lower than 0.2 in the univariate analysis [32] were entered into a final model of multivariable analysis as independent variables. Age, insurance status, and household size had significance levels more than 0.2 and were excluded and 95% confidence intervals for adjusted odds ratios were reported.

All data manipulation and analyses and graphics to display geographical disparities across provinces were conducted with STATA software version 12.0 (Stat Corp. LP, College Station, TX, USA) and ArcMap 10.1, respectively. We displayed the variation of CHE disparities across provinces by quintile of provinces from first to fifth quintile (i.e. each quintile shows one fifth of the total number of provinces). The analyses were done at household level. Sampling weight, resulting from the method of sampling, was entered into all analyses.

**Table 1:** Sample size for the study years from 2008 to 2015

Year	Residency	Primary sample	Excluded sample^a^	Final sample	Sample size
2008	Urban	19,381	43	19,338	39,008
	Rural	19,707	37	19,670	
2009	Urban	18,665	59	18,606	36,772
	Rural	18,203	37	18,166	
2010	Urban	18,701	52	18,649	38,176
	Rural	19,584	57	19,527	
2011	Urban	18,727	32	18,695	38,434
	Rural	19,786	47	19,739	
2012	Urban	18,535	33	18,502	38,117
	Rural	19,657	42	19,615	
2013	Urban	18,880	46	18,834	38,244
	Rural	19,436	26	19,410	
2014	Urban	18,885	28	18,857	38,191
	Rural	19,390	56	19,334	
2015	Urban	18,871	45	18,826	38,148
	Rural	19,381	59	19,322	
Sum	Urban	131,774	293	131,461	305,090
	Rural	135,763	302	135,479	

^a^Number of households with no food expenditure report

## Results

Findings presented in Table 2 show that the total expenditure of households had increased over the study period; it was 2.5 times higher in 2015 compared to that of 2008. Findings also revealed that households’ CTP had increased in rural and urban areas and it was more than 2.4 times higher in 2015. These increases were almost the same in both rural and urban areas.

**Table 2:** Total consumption expenditure, food expenditure, capacity to pay, poverty line, and health expenditure from 2008 to 2015 (Numbers in thousand rials^a^)

Year	Residency area	Total consumption expenditures^b^	Food expenditures^b^	Capacity to pay^b^	Health expenditure^b^ (OOP)^c^	Average poverty line^d^
2008	Urban	8102	1696	6801	494	810
	Rural	4604	1692	3365	300	727
	Total	7084	1695	5780	437	812
2009	Urban	8563	1809	7147	563	899
	Rural	5049	1786	3693	394	775
	Total	7601	1802	6168	517	912
2010	Urban	9736	2097	8092	703	1042
	Rural	5830	2130	4258	444	954
	Total	8699	2107	7079	634	1011
2011	Urban	11,256	2634	9110	595	1334
	Rural	7100	2695	5032	401	1203
	Total	10,128	2650	8006	542	1293
2012	Urban	13,973	3599	11,170	746	1722
	Rural	9162	3736	6207	529	1829
	Total	12,730	3635	9845	690	1800
2013	Urban	17,571	4430	14032	1176	2231
	Rural	10,966	4582	7333	749	2233
	Total	15,818	4471	12,211	1062	2287
2014	Urban	20,032	4669	16,303	1341	2354
	Rural	11,749	4673	8019	781	2223
	Total	17,857	4670	14,105	1194	2393
2015	Urban	22,319	5020	18,333	1597	2289
	Rural	12,458	4737	8779	855	2525
	Total	19,756	4946	15,774	1404	2566

^a^The currency rate of 1 PPP $ (Purchasing power parity/International Dollar) in study years were as follows: 2008 = 3058.0, 2009 = 3133.5, 2010 = 3525.4, 2011 = 4809.1, 2012 = 5472.2, 2013 = 8434.0, 2014 = 8595.7 and 2015 = 8369.5; ^b^ Per-household per-month; ^c^ Out-of-pocket payment; ^d^ Poverty line per-person per-month

There was also a remarkable difference in households’ total expenditures between rural and urban areas in all the studied years. The total expenditure of an urban household was 52% to 76% higher than that of a rural household. Furthermore, CTP of every urban family was at least 80% higher than that of a rural family.

Average food expenditure had increased for every household over the years; it was more than 2.8 times higher in 2015. The increase was relatively similar in urban and rural areas.

Findings also showed that the monthly average of PL per person had increased in all the study years in both urban and rural areas; it was about 3 times higher in 2014 and 2015. The average absolute monetary value of PL (per-person, per-month) in rural areas was less than that in urban areas, with highest difference observed in 2014 (130,504 Rials).

Monthly OOP payments were higher in urban areas. Compared with the baseline year (2008), the OOP payments in 2015 had a relatively remarkable increase. In addition, health expenditures had an increase in both urban and rural areas over the years, except for 2011. More details about total consumption expenditure, food expenditure, and CTP of households are illustrated in Table 2.

As it is shown in Table 2, in terms of a monetary value, the OOP payments and poverty line were higher in 2014 and 2015. In fact, although there was an increase in income and CTP, but the increase in the OOP payments was relatively higher than the income and CTP, leading to the observed differences in these two years.

**Fig. 1:**
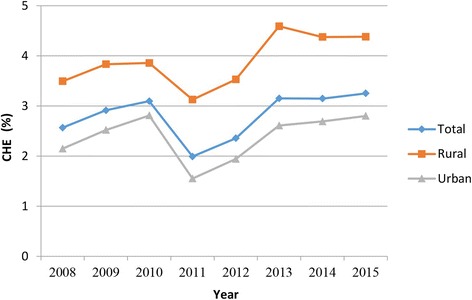
CHE prevalence (%) in Iran by residence area from 2008 to 2015

Figure 1 shows the percentage of households who experienced CHE based on CTP in both rural and urban areas. The exposure rate had relatively increased over the years; it increased from 2.57% in 2008 to 3.25% in 2015. However, there was a decreased in CHE rate from 3.1% in 2010 to 1.99% in 2011.

As shown in Table 3, the intensity of CHE (i.e. the ‘overshoot’ at 40% threshold) ranged from 0.26% to 0.65% over the study years. Generally, the overshoot was similar (about 0.42%) for 2014 and 2015 (years after IHTP implementation in early 2014). However, in terms of MPO (i.e. expenditure beyond the threshold), these rates for the households that actually experienced catastrophe at 40% threshold ranged from 12.26% to 20.86% in the study years. The MPO in 2014 and 2015 (i.e. years after IHTP adoption) was 13.5% and 12.88%, respectively.

**Table 3:** Prevalence and intensity of catastrophic health expenditures by residence area from 2008 to 2015

Year	Index	Residency area		Total
		Rural	Urban	
2008	CHE %^a^ (95% CI)^b^	3.49 (3.26–3.91)	2.15 (1.87–2.42)	2.57 (2.34–2.78)
	Overshoot %	0.57	0.38	0.44
	MPO %	16.45	17.69	17.25
2009	CHE % (95% CI)	3.83 (3.58–4.31)	2.52 (2.08–2.95)	2.91 (2.57–3.24)
	Overshoot %	0.82	0.47	0.58
	MPO %	21.35	18.87	19.83
2010	CHE % (95% CI)	3. 86 (3.56–4.22)	2.81 (2.48–3.13)	3.09 (2.84–3.34)
	Overshoot %	0.66	0.67	0.65
	MPO %	17.21	23.76	20.86
2011	CHE % (95% CI)	3.13 (2.6–3.07)	1.55 (1.39–1.75)	1.99 (1.80–2.17)
	Overshoot %	0.34	0.21	0.26
	MPO %	11.02	13.63	12.94
2012	CHE % (95% CI)	3.53 (3.19–3.84)	1.94 (1.68–2.21)	2.36 (2.13–2.57)
	Overshoot %	0.41	0.24	0.29
	MPO %	11.65	12.35	12.26
2013	CHE % (95% CI)	4.59 (4.23–4.98)	2.61 (2.32–2.92)	3.15 (2.90–3.39)
	Overshoot %	0.60	0.37	0.44
	MPO %	13.11	14.05	14.00
2014	CHE % (95% CI)	4.38 (4.02–4.72)	2.69 (2.40–3.01)	3.15 (2.90–3.39)
	Overshoot %	0.61	0.33	0.42
	MPO %	14.06	12.33	13.50
2015	CHE % (95% CI)	4.38 (4.03–4.74)	2.81 (2.50–3.14)	3.25 (3.00–3.51)
	Overshoot %	0.59	0.35	0.42
	MPO %	13.57	12.43	12.88

^a^Prevalence of CHE ^b^ 95% confidence interval (CI)

**Fig. 2:**
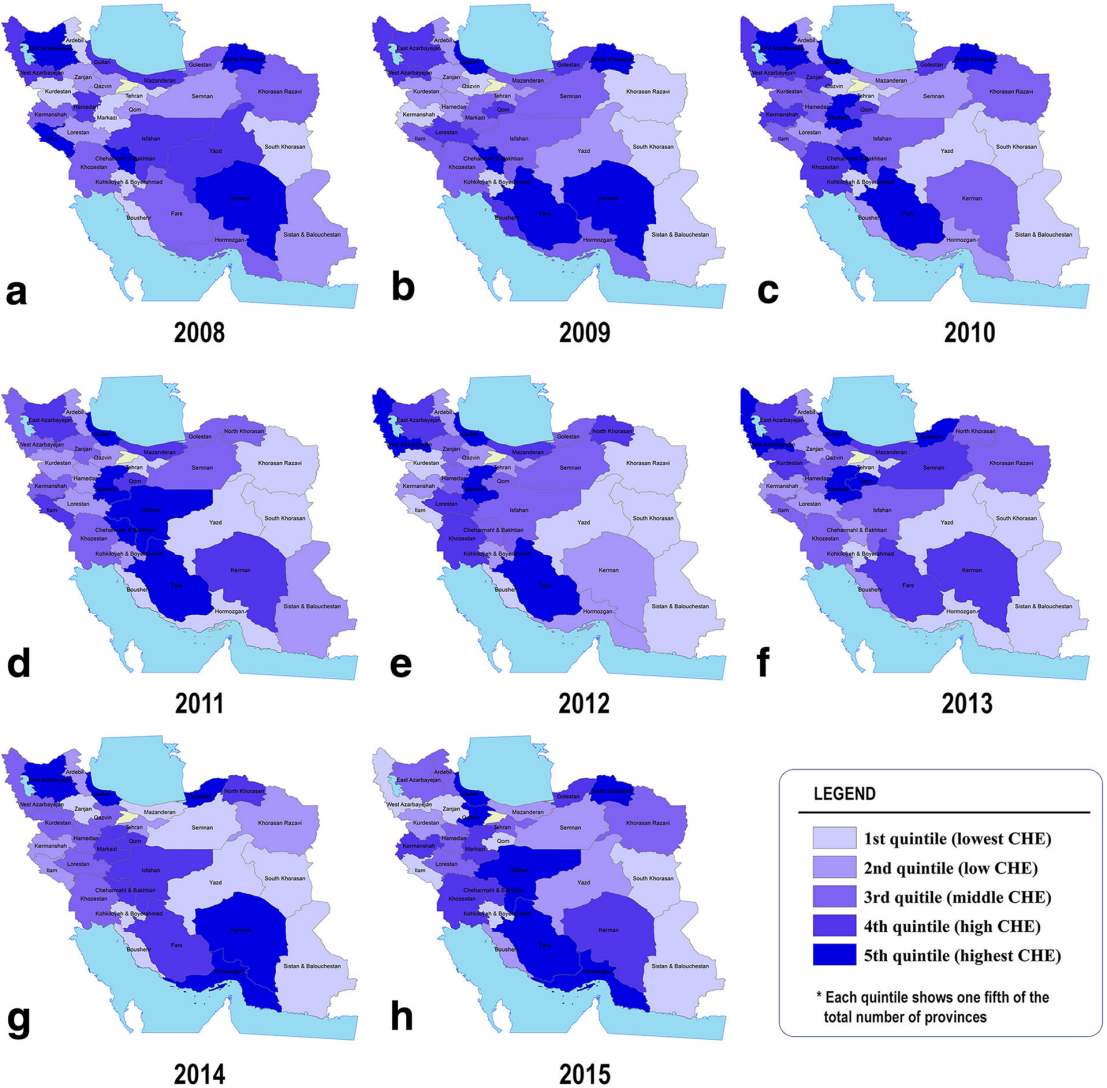
Provincial disparity in households facing CHE (%) from 2008 to 2015 (left to right)

Figure 2 shows the provincial differences in percentage of households who experienced CHE during the study years. The findings revealed that, on average, Fars, Guilan and Markazi provinces had the highest percentage of households facing CHE over the years. As shown in Fig. 2, provinces located in east and eastern south, such as Khorasan-E-Jonoubi and Sistan & Balouchestan, had the lowest percentage of CHE prevalence in most of the study years. Interestingly, these provinces are of Iranian provinces with limited geographical and cultural accessibility.

As shown in Table 4, determinants of catastrophic health expenditure (CHE) prevalence were assessed by a multivariable logistic random effects regression model. The results showed that in most of study years, there was significant relationship between CHE experience and household place of residence (rural, urban), household income, experience of inpatient healthcare, outpatient healthcare, and presence of elderlies living in the household (*p* < 0.05). Interestingly, there were no significant relationship between CHE experience and gender of head of households (*p* > 0.05).

**Table 4:** Determinants of catastrophic health expenditure from 2008 to 2015 using a logistic random effects regression model

Variable	Year/ adjusted OR (95% CI)							
	2008	2009	2010	2011	2012	2013	2014	2015
Expenditure quintiles Q1(poorest) Q2 Q3 Q4 Q5(richest)	Referent 0.57 (0.48–0.67)^a^ 0.46 (0.38–0.57)^a^ 0.41 (0.31–0.56)^a^ 0.31 (0.18–0.51)^a^	Referent 0.71 (0.59–0.85^a^ 0.52 (0.39–0.68)^a^ 0.40 (0.30–0.54)^a^ 0.32 (0.19–0.54)^a^	Referent 0.54 (0.46–0.62)^a^ 0.42 (0.35–0.51)^a^ 0.36 (0.28–0.46)^a^ 0.40 (0.28–0.58)^a^	Referent 0.71 (0.56–0.90)^a^ 0.45 (0.33 0.61)^a^ 0.32 (0.23–0.46)^a^ 0.32 (0.22–0.48)^a^	Referent 0.77 (0.61–0.96)^a^ 0.47 (0.36–0.63)^a^ 0.38 (0.29–0.50)^a^ 0.32 (0.22–0.47)^a^	Referent 0.73 (0.60–0.89)^a^ 0.50 (0.38–0.65)^a^ 0.43 (0.33–0.57)^a^ 0.36 (0.26–0.49)^a^	Referent 0.73 (0.59–0.90)^a^ 0.52 (0.40–0.67)^a^ 0.31 (0.25–0.39)^a^ 0.23 (0.17–0.32)^a^	Referent 0.72 (0.58–0.87)^a^ 0.52 (0.39–0.64)^a^ 0.47 (0.36–0.57)^a^ 0.49 (0.33–0.63)^a^
Household settlement Urban Rural	Referent 1.36 (1.11–1.65)^a^	Referent 1.19 (1.03–1.37)^a^	Referent 1.06 (0.95–1.18)	Referent 5.63 (3.94–8.05)^a^	Referent 1.34 (1.13–1.59)^a^	Referent 1.36 (1.19–1.55)^a^	Referent 1.32 (1.16–1.50)^a^	Referent 1.35 (1.15–1.51)^a^
Household head gender Male Female	Referent 1.02 (0.79–1.21)	Referent 1.36 (1.14–1.62)^a^	Referent 1.17 (1.01–1.35)^a^	Referent 1.02 (0.83–1.26)	Referent 1.12 (0.94–1.33)	Referent 1.07 (0.93–1.23)	Referent 1.03 (0.84–1.27)	Referent 1.01 (0.79–1.20)
Hospitalized person in Household No Yes	Referent 1.72 (1.62–1.83)^a^	Referent 3.15 (2.65–3.75)^a^	Referent 2.80 (2.28–3.46)^a^	Referent 1.71 (1.54–1.90)^a^	Referent 1.48 (1.29–1.69)^a^	Referent 0.96 (0.73–1.26)	Referent 1.33 (1.24–1.43)^a^	Referent 1.75 (1.41–2.03)^a^
Household using outpatient care No Yes	Referent 1.69 (1.47–1.85)^a^	Referent 3.53 (2.71–4.59)^a^	Referent 5.66 (4.62–6.94)^a^	Referent 7.27 (4.52–11.68)^a^	Referent 10.05 (7.08–14.29)^a^	Referent 5.76 (4.50–7.38)^a^	Referent 3.04 (2.32–3.98)^a^	Referent 5.25 (4.15–6.91)^a^
+ 60 member living in Household No Yes	Referent 1.88 (1.56–2.26)^a^	Referent 1.68 (1.40–2.02)^a^	Referent 1.63 (1.35–1.97)^a^	Referent 2.18 (1.76–2.71)^a^	Referent 1.83 (1.46–2.29)^a^	Referent 1.91 (1.59–2.28)^a^	Referent 1.01 (0.89–1.15)	Referent 2.09 (1.72–2.39)^a^
Statistics	Wald-chi2: 709.8 Pseudo R2 = 0.16	Wald-chi2: 550.9 Pseudo R2 = 0.17	Wald-chi2: 537.6 Pseudo R2 = 0.17	Wald-chi2: 485.8 Pseudo R2 = 0.07	Wald-chi2: 377.4 Pseudo R2 = 0.12	Wald-chi2: 483.9 Pseudo R2 = 0.09	Wald-chi2: 743.4 Pseudo R2 = 0.11	Wald-chi2: 657.6 Pseudo R2 = 0.13

^a^The odds ratios that became significant after adjustment for cluster (Province) effect

## Discussion

This study aimed to measure the prevalence and intensity of CHE over a seven-year period (2008–2015) in urban and rural areas in Iran. We also explored the disparities in CHE by geographical regions and revealed the factors that affect CHE prevalence.

The overall rate of households facing CHE increased over the study years, with an average growth rate of 2.5%. As with the present study, a study conducted in national level in Iran, that used the same surveys and methods and spanned from 2003 to 2007 [33], also showed that the CHE rate ranged from 2.25% to 2.5% in that period. A study in Egypt also showed an upward CHE trend from 2000 to 2010, but mean of CHE was about 6% and higher than that of Iran [34]. In another study conducted in one of Burkina Faso states, with a similar threshold level, the rate of households facing CHE was 8.66%, much higher than the overall rate observed in the present study [4]. Such a difference can be due to different population characteristics, poverty rate, income and economic status in countries. In a study carried out by Xu et al. on 59 countries, it was shown that the rate of households facing CHE ranged from 0.01 in Czech Republic and Slovakia to 10.5% in Vietnam [5]. However, due to establishment of tax-based healthcare financing system, developed social insurance institutions, and pro-poor policies the rate of households facing CHE was very low in most of developed countries. However, this rate was more than 3% [5] in developing countries, as with Iran. Developing countries like Vietnam, Cambodia, Azerbaijan, Ukraine, and Latin American countries such as Argentina, Brazil, Colombia, Paraguay, and Peru had the highest rates of households facing CHE [5]. This can be due to reasons like certain economic structures, pre-paid mechanisms for social protection, and the OOP spending proportion.

In our study, the overshoot of CHE ranged from 0.26% to 0.65% (mean = 0.34%). It means that, on average, households spent 0.34% over the 40% catastrophic threshold. The households that actually had experienced catastrophe at 40% threshold spent 12.26% to 20.86% (MPO) over the threshold. Thus, these households spent 52.26% to 60.86% (40% threshold + MPO) of CTP on OOP. Both overshoot and MPO, as intensity measures, did have an oscillatory trend during study years. However, compared to baseline year (i.e. 2008), these two measures showed little change after IHTP implementation. A cross-country study conducted in Egypt, Jordan, Palestine [34] (with 40% threshold) showed that the overshoot ranged from 0.1% to 0.8% from 2000 to 2010. Moreover, MPO in that study ranged from 11.1% to 16.2% over the same period of time [34]. All these findings are in line with our study.

In order to lessen CHE rates, the national development plans in Iran propose some reforms such as development of effective referral systems based on family physician program and integration of fragmented insurance funds [14]. Nevertheless, primary health care (PHC) and the referral system have remained a challenge in urban areas [35]. This matter has led to some healthcare system dysfunctions and unnecessary spending. Integration of insurance funds has not been also realized yet [36]. This matter has led to inequitable access to benefit packages, co-payments, and limited financial protection against medical expenditures among different groups of population. WHO postulates that when the share of OOP spending decreases by 15 to 20%, the proportion of households facing CHE will be negligible [10].

However, a high CHE prevalence can also be caused by high OOP spending resulting from improvements in health care accessibility. Furthermore, an increase in CHE prevalence might also be due to increased PL emanating from higher inflation rates. For instance, as showed in a study by Zare et al., inflation rate had higher effects on health expenditures of the poor than the rich [37]. Therefore, one should be cautious when discussing about high CHE rates.

In general, geographic disparities in the rate of households facing CHE in rural and urban areas had increased over the study period. The average CHE prevalence was higher in rural areas, as with other studies [38–40]. Owing to remarkable inequalities in socio-economic development in rural and urban regions of Iran, households’ CTP, average income, and poverty line differ across the areas. Therefore, the gap between households’ CTP in rural and urban areas can be due to more low-income households in rural areas [41]. According to the findings, health expenditures had increased during the study years in both urban and rural areas. However, health expenditures of rural households were lower than urban households which can be due to implementation of rural family physician program and the referral system in rural areas. Moreover, health-seeking behavior, which can arise from cultural differences in these areas, might also affect such differences in health expenditures between rural and urban areas. Zare et al. have revealed that there have been increasing inequalities in health expenditures in Iran, especially between urban and rural areas, over the past three decades [37]. Similarly, other studies have revealed that healthcare expenditures are higher in rural areas in Iran [33, 42].

According to findings, there was significant variation across provinces in terms of CHE rate. However, some counterintuitive cases of CHE rate in provinces should be scrutinized carefully. For example, the least developed provinces such as Sistan & Balouchestan or newly announced provinces like Birjand and Alborz had the least CHE prevalence. This matter could be due to some factors outside health system, such as lower income per capita and lower education, or factors within health system, like lower geographical and cultural access to health services. These factors could limit health services utilization in these regions [43]. In addition, the implementation of IHTP in early 2014 might have increased inpatient services in the public sector in those regions. In contrast, more developed provinces such as Fars had higher CHE rate than other provinces of Iran. These findings are compatible with those of Kavosi et al. study in Shiraz [19], the capital of Fars province. These provinces have relatively high education and income per capita and higher access to more specialized health services providers. Interestingly, some developed provinces such as Tehran, capital of Iran, had low rate of CHE prevalence. However, considering their huge population, the absolute number of households facing CHE is high in such provinces. A study conducted in district 17 of Tehran [16], one of the least developed areas in the city, showed that the percentage of households facing CHE in 2003 and 2008 was 12.6% and 11.8%, respectively.

Upon implementation of IHTP in early 2014, remarkable financial resources were allocated to inpatient medical services. This led to a rapid increase in share of government and insurance funds in healthcare financing. In fact, our findings showed that two years after IHTP implementation, CHE prevalence was higher than the rate in 2008 and equal to the rate in the preceding year. Some reasons can account for this unexpected finding about the CHE prevalence. First, increased utilization of health services that resulted from unmet needs in the previous years. Second, more focus on inpatient services in public sector in the IHTP. Third, despite an increase in the share of government and insurance funds in total health expenditures, remarkable rise in health tariffs, with a relative value unit (RVU) for health services, increased the OOP payments of the households even more than the previous years. However, the IHTP was able to reduce the intensity of CHE, particularly the MPO, for households that actually experienced catastrophe. Nevertheless, despite attempts to reduce the *OOP* spending, as a percentage of total health expenditure [44], the IHTP failed to realize it.

Although provision of free access to basic health insurance for all is an effective effort to move toward universal health coverage, it led to more unexpected coverage of people in IHTP without having a suitable mechanism to distinguish disadvantaged individuals. This has put pressure on insurance funds and challenged the resource sustainability. Strangely, some studies have shown that despite implementation of health insurance schemes, the insured persons still remain vulnerable to medical expenses [45]. Therefore, although health insurance can reduce the burden of financial catastrophes [46, 47], its coverage capacity, committed services, and covered costs should be considered in health policy making. For instance, a financial protection system was deployed to reduce the CHE prevalence among disadvantaged groups in Georgia when they adopted a similar program [44].

Rural residence, lower income, presence of an elder member in household, and receiving inpatient and outpatient services increased the probability of experiencing CHE. This is consistent with results reported by some other studies such as Yardim et al. in Turkey [48], Kavosi et al. in Iran [15], and Galárraga et al. in Mexico [46]. Our study indicated that rural residence increases the probability of catastrophic health spending by households. A possible explanation for this might be that rural people have lower income and CTP, lower education, and less access to comprehensive health services while they suffer from more illnesses. Another important finding was that presence of elderlies’ household increase the probability of household CHE. In this regard, it can be noted that a longer life span increases the probability of emerging new and costly non-communicable diseases (e.g. Musculoskeletal, neurological and dementia diseases) [49]. Furthermore, it is interesting to note that the odds ratios of outpatient services were higher than inpatient/hospitalization services. This might indicate that proportion of the costs covered by basic health insurance in outpatient services is lower than inpatient ones. Overall, it can be also concluded that during study period, particularly by implementation of IHTP, achieving equal access to services and basic health insurance coverage were improved; but stronger risk protection, strategic and proactive purchasing, greater efficiency and quality of care based on moving toward to UHC are still remained as challenges. Thus, in addition to breadth of insurance coverage as suggested by WHO report [10], insurance policies that target poverty and rural areas and purposeful service packages for at risk groups such as elder populations might be of help in reducing the CHE rate in Iran. Interestingly, odds ratios of these variables were statistically insignificant and diverging from the general pattern in some years. This can be due to the fact that the data are random and they do not have the same yearly ratio. In addition, such an inconsistency can be affected by different patterns of inpatient services in the study years or by recall bias of respondents.

### Study limitations

This study had some limitations. First, although the method used is one of the most common methods, but the households who could not afford to pay for health care services are not addressed in the CHE methodology. This matter can somehow cloud the CHE measurement results. Second, the used measures do not consider indirect expenses imposed on households, such as travel costs and decreased or loss of incomes due to illness. Finally, although we tracked the CHE prevalence for two years after IHTP implementation, it may not suffice to give reliable evidence of IHTP effects on CHE and further studies with more subtle designs are strongly required. All these limitations should be borne in mind when interpreting the findings.

## Conclusions

The present study showed that the rate of households experiencing CHE had increased over the study period and it was higher in rural areas. The geographic inequalities across provinces had also worsened over the period. Like many middle-income countries, health services have been extended in Iran over the past decades. However, the main health policies implemented in previous years, particularly implementation of the IHTP in early 2014, have not yet realized the objectives like lessening the proportion of households experiencing CHE to 1.0%, endorsed by national development plans. Moreover, rural households were more vulnerable to CHE than urban households and their capacity to pay was low. It should be noted that provinces had different CHE rates which might be affected by different features of the cultural and socio-economic factors. These differences should be addressed by applying tailored and context-sensitive policies. A high priority therefore is with the plans that aim to revise health care protective financing. Those plans can focus on pre-payments, interventions that target disadvantaged population, extension of health services coverage, and efforts to eliminate unemployment and poverty based on regional differences.

## Abbreviations

CHE: Catastrophic Health Expenditures
CI: Confidence Interval
CTP: Capacity to pay
IHTP: Iran’s Health Transformation Program
ISC: Iran Statistics Center
OOP: Out-of-Pocket
SSO: Social Security Organization
UHC: Universal Health Coverage
